# Potential Therapeutic Effects of Oolong Tea Phytochemicals on NLRP3 Inflammasome Assembly and Oxidative Stress

**DOI:** 10.3390/nu17193106

**Published:** 2025-09-30

**Authors:** Ming-Shyan Wang, Szu-Nian Yang, Yi-Ping Chang, Chi-Sheng Wu, Hung-Chi Yang, Jia-Feng Chang

**Affiliations:** 1 Division of Clinical Pharmacy, Department of Pharmacy, Far Eastern Memorial Hospital, New Taipei 220216, Taiwan; mingshya@mail.femh.org.tw; 2 Department of Psychiatry, Taipei Veterans General Hospital, Taoyuan Branch, Taoyuan 330023, Taiwan; ysn56725@ms4.hinet.net; 3 Department of Oral Hygiene, Jen-Teh Junior College of Medicine, Nursing and Management, Miaoli 356006, Taiwan; 4 Healthcare Information and Management, Ming Chuan University, Taoyuan 333321, Taiwan; D228@tyvh.gov.tw; 5 Division of Nephrology, Department of Internal Medicine, Taipei Veterans General Hospital, Taoyuan Branch, Taoyuan 330023, Taiwan; 6 Renal Care Research and Health Promotion Association, New Taipei 220050, Taiwan; luke.chisheng@gmail.com; 7 Department of Medical Laboratory Science and Biotechnology, Yuanpei University of Medical Technology, Hsinchu 300102, Taiwan; hcyang@mail.ypu.edu.tw; 8 Department of Nursing, Yuanpei University of Medical Technology, Hsinchu 300102, Taiwan

**Keywords:** oolong tea phytochemicals, NLRP3, ROS, ASC, Caspase-1, IL-1β

## Abstract

Background/Objectives: Tea, the world’s second most consumed beverage after water, contains diverse phytochemicals that have garnered growing interest for their potential ability to modulate inflammasome activation. This study examined the antioxidant and anti-inflammatory properties of oolong tea (OLT) extracts, with a specific focus on their regulatory effects on NLRP3 inflammasome assembly—a critical mediator in chronic inflammatory diseases. Methods: OLT extracts were prepared from the Jin-Xuan cultivar with quantification for bioactive components (total phenolics, flavonoids, condensed tannins, and proanthocyanidins). J774A.1 murine macrophages were primed with LPS and stimulated with ATP to induce inflammasome activation. Therapeutic potentials of OLT extracts were assessed by measuring cytokine secretion, expression of NLRP3 inflammasome-related proteins (NLRP3, ASC, Caspase-1, and IL-1β), inflammasome complex formation, and ROS generation via biochemical assays, immunoblotting, and fluorescence microscopy. Results: OLT extracts, particularly at 100 µg/mL, markedly suppressed both the priming and activation phases of NLRP3 inflammasome formation. OLT treatment reduced IL-1β secretion by more than 50%, attenuated ASC oligomerization and speck formation, inhibited caspase-1 cleavage, and lowered intracellular ROS levels by approximately 50%. Conclusions: These findings suggest that OLT extracts exert potent anti-NLRP3 inflammasome activity and offer immunomodulation potential in preventing inflammation-related diseases such as infections, cancer, and neurodegenerative disorders. Further in vivo investigations, followed by clinical applications and epidemiological studies, are warranted to validate these preventive effects in human populations.

## 1. Introduction

Tea, derived from the plant *Camellia sinensis*, is one of the most popular beverages consumed in the world, with around 6.7 million tonnes produced annually [1]. Oolong tea (OLT) accounts for about 2% of global tea production and is partially fermented—undergoing 10–70% oxidation—which gives it its distinctive flavor and aroma [2,3]. OLT originated in Fujian Province, China, and has subsequently become an integral element of Taiwanese tea culture. It is valued for its rich content of polyphenols (such as gallic acid and proanthocyanidins), catechins (including EGCG, EGC, ECG, and EC), and volatile compounds (such as linalool, geraniol, and nerolidol) [4,5]. These bioactive components contribute to various health benefits, including anti-tumor effects, anti-microbial activity, immune modulation, boosting metabolism, anti-diabetes, anti-allergic, anti-inflammatory, cardioprotective and anti-hypertensive properties [6,7,8,9].

Studies have shown that oolong tea polyphenols (OLPs) effectively scavenge reactive oxygen species (ROS), thereby reducing oxidative stress and lipid peroxidation. This protective action against DNA damage contributes to a lower risk of chronic diseases like cancer and age-related neurodegeneration [10]. OLT also exhibits stronger antimutagenic effects than green or black tea, with polyphenols inhibiting cancer cell invasion, inducing apoptosis, and causing cell cycle arrest [6,11,12]. Notably, OLT’s anti-inflammatory activity reportedly surpasses that of green and black teas; its catechins, tannins, and theaflavins, formed during partial fermentation, are considered key to these effects due to their role in reducing oxidative damage and cancer-promoting inflammation [12,13].

With varying degrees of fermentation, tea undergoes complex chemical transformations that result in the formation of unique compounds such as thearubigins, theaflavins, and theasinensins. Theaflavins and theasinensins, in particular, have been reported to exhibit anti-inflammatory effects in several studies [14,15]. Furthermore, unique OLPs known as theasinensins, specifically theasinensin A (TSA), a product formed from the oxidation of epigallocatechin gallate (EGCG) [16], has been shown to inhibit key inflammatory mediators such as interleukin-12 (IL-12) and tumor necrosis factor-alpha (TNF-α). TSA also exhibits antioxidative effects, induces apoptosis, and inhibits matrix metalloproteinases, highlighting its broader potential in chemoprevention [17,18].

Although rare, allergic reactions to unfermented teas such as green tea have been reported, often linked to sensitization to EGCG [19,20,21]. High levels of EGCG may also cause mild side effects, including bloating, nausea, and dizziness [22]. In contrast, OLT contains approximately twice the amount of polymerized polyphenols and only half the EGCG found in green tea, potentially delivering similar health benefits with a lower risk of allergic response [23,24]. Moreover, while high concentrations and frequent intake of tannins can lead to digestive discomfort, OLT contains slightly less tannin than black tea, making it a gentler alternative for individuals with sensitive digestive systems [25]. Despite its popularity, the health-promoting effects of oolong tea and its unique bioactive compounds remain relatively understudied compared to green tea [26].

The NLR family pyrin domain containing protein 3 (NLRP3) inflammasome, a key multiprotein complex in the innate immune system, regulates inflammation via its NLRP3 sensor, ASC (apoptosis-associated speck-like protein containing a caspase recruitment domain) adaptor, and caspase-1 effector components [27]. It activates inflammatory responses to infection and stress, but excessive activation can drive chronic inflammation, linked to diseases such as rheumatoid arthritis, type 2 diabetes, gout, metabolic dysfunction-associated steatohepatitis, atherosclerosis, Alzheimer’s disease, inflammatory bowel disease, etc. [28,29]. OTPs, particularly catechins, exhibit anti-inflammatory and antioxidant effects that may reduce NLRP3 inflammasome activity by scavenging ROS, suggesting their therapeutic benefits for inflammasome-driven diseases [30,31,32].

**Figure 1:**
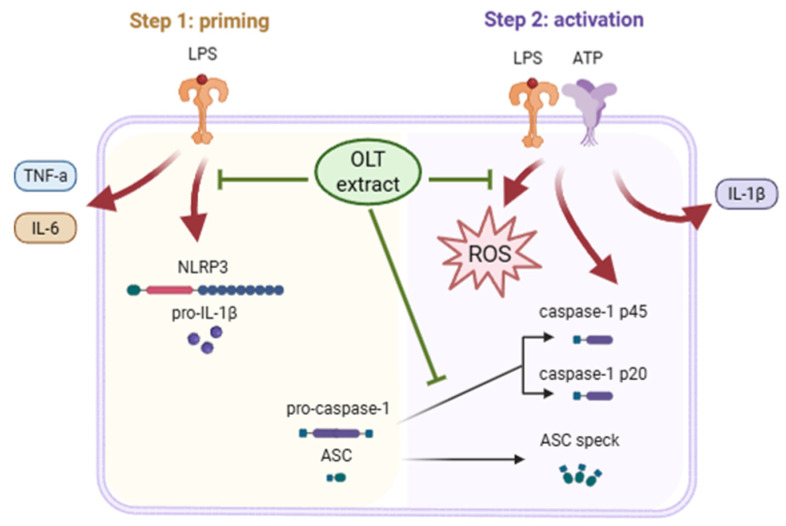
Graphical Abstract: Across priming and activation phases, OLT decreases IL-6/TNF-α and key inflammasome readouts while lowering intracellular ROS. Created in Biorender. Chi-Sheng Wu. (2025) https://BioRender.com/.

According to the above evidence-based literature, the potential immunomodulatory effects of OLT phytochemicals on NLRP3 inflammasome pathways remain elusive. Lipopolysaccharides (LPS) derived from a major component of the outer membrane of Gram-negative bacteria is a potent immunostimulant to activate macrophages. Thus LPS-stimulated macrophages serve as a commonly used in vitro model to mimic aspects of bacterial infection and the inflammatory response it triggers [33]. Figure 1 briefly illustrates the proposed therapeutic mechanism of OLT extract in modulating various notable markers of NLRP3 inflammasome pathways. In the context of the NLRP3 inflammasome process, “priming,” “assembly,” and “activation” refer to distinct stages of inflammasome function. Priming stage: Pro-inflammatory signals (LPS) activate the nuclear factor kappa B (NF-κB) pathway, inducing the transcription of NLRP3, pro-interleukin-1β (IL-1β), and pro-Caspase-1. This stage also promotes the secretion of cytokines like interleukin 6 (IL-6) and TNF-α, priming the cell for subsequent inflammasome assembly without full activation [27,34]. Assembly stage: A secondary signal, such as adenosine triphosphate (ATP) or ROS, triggers NLRP3 oligomerization. NLRP3 recruits ASC, which then binds pro-Caspase-1, forming the inflammasome complex. Activation stage: The assembled inflammasome activates Caspase-1, which cleaves pro-IL-1β into its active form, IL-1β, leading to its secretion and initiating the inflammatory responses [27,29,35].

Collectively, based on health benefits and cultural significance in Asia, OLT was selected to investigate its influence on regulatory factors in LPS-stimulated macrophages. While OLT has known anti-inflammatory and antioxidant properties, its potential immunomodulatory effects on the NLRP3 inflammasome remain to be further elucidated. This study aims to clarify the mechanisms by which OLT modulates NLRP3, potentially informing new therapeutic strategies.

## 2. Materials and Methods

### 2.1. Material and Reagents

(+)-catechin, (−)-gallocatechin, (−)-epigallocatechin, (−)-epicatechin, (−)-epigallocatechin gallate, (−)-gallocatechin gallate, (−)-epicatechin gallate, (−)-catechin gallate, (+)-catechin hydrate, cyanidin chloride, caffeine and gallic acid standards were purchased from Sigma-Aldrich (St. Louis, MO, USA). HPLC-grade acetonitrile, acetic acid, 0.1% (*v*/*v*) Formic acid in water, and acetonitrile with 0.1% (*v*/*v*) formic acid were obtained from Sigma-Aldrich (St. Louis, MO, USA). RPMI-1640 medium, fetal bovine serum (FBS), L-glutamine, Disuccinimidyl suberate (DSS), loading buffer, LPS, ATP, and Invitrogen SDS-PAGE Gels were purchased from ThermoFisher Scientific (Waltham, MA, USA). Folin–Ciocalteu reagent, vanillin, sodium carbonate, sodium nitrite, aluminum chloride, sodium hydroxide, CHAPS buffer, Tris-buffered saline (TBS), fluorescent probe 2′,7′-dichlorofluorescein diacetate (DCFH-DA) and other reagent-grade chemicals used in the analysis were obtained from Sigma-Aldrich (St. Louis, MO, USA). Murine macrophages (J774A.1 cell line) were obtained from the American Type Culture Collection (Rockville, MD, USA).

### 2.2. Tea Material

The Taiwan Tea Experiment Station No. 12 (TTES No. 12), commonly known as the Jin Xuan cultivar, was used in this study. This cultivar is widely applied in the processing of oolong tea and was obtained from the Tea Research and Extension Station in Taiwan (see official source: https://www.tbrs.gov.tw/en/ (accessed on 22 November 2024)). Tender shoots, consisting of the top two leaves and a bud, were harvested, freeze-dried (Yamato Freeze Dryer DC 400, Tokyo, Japan) to constant weight, and then ground into powder using a grinder (RT-02, Rong Tsong Iron Factory, Taipei, Taiwan). The resulting powder was stored at –20 °C or directly used for polyphenol extraction.

### 2.3. Extraction

To mimic the traditional brewing process of oolong tea, TTES No. 12 (Jin Xuan) powder was extracted twice with hot water (95 °C, 1:50 ratio) and shaken vigorously for 60 min. The resulting filtrate was freeze-dried to obtain dry powder, which was designated as OLT and used for subsequent phytochemical characterization, including determination of total phenolics, flavonoids, condensed tannins, and proanthocyanidins [36]. We do not currently have quantitative measurements of solubility or long-term stability of the OLT powder or reconstituted extract. The concentrations used in this work are within the range reported in previous anticancer and bioactivity studies [3] and were sufficiently dissolved under the conditions of our assays. Stability beyond the assay period, under varied storage conditions, remains to be further investigated.

### 2.4. Determination of Total Phenolic Content

The total phenolic content was measured using a colorimetric assay modified from Luximon-Ramma et al., (2002) [37]. For this assay, OLT extracts were freshly dissolved in distilled water at a concentration of 1 mg/mL. Briefly, 50 μL sample was mixed with 1 mL distilled water, 0.5 mL Folin–Ciocalteu reagent, and 2.5 mL sodium carbonate (20%), and then left to react in darkness for 20 min. The coloration was developed, and the absorbance was measured at 735 nm (BioTek Epoch Microplate Spectrophotometer, Agilent Technologies, Santa Clara, CA, USA). Results were expressed as mg gallic acid equivalents (GAE) per gram of dry sample weight, with a gallic acid standard curve (0–1000 μg/mL).

### 2.5. Determination of Total Flavonoid Content

Flavonoid content was determined by the aluminum chloride method (Sakanaka et al., 2005) [38]. For this assay, OLT extracts were freshly dissolved in distilled water at 1 mg/mL. A 250 μL sample was mixed with 1.25 mL distilled water and 75 μL of sodium nitrite (5%) and allowed to react for 6 min. Then, 150 μL of aluminum chloride (10%) was added, followed by 500 μL sodium hydroxide (1 M) and 2 mL distilled water. The absorbance was recorded at 510 nm using a spectrophotometer. Flavonoid content was expressed as mg catechin equivalents (CE) per gram of dry weight, with a (+)-catechin hydrate standard curve (0–1000 μg/mL).

### 2.6. Determination of Total Condensed Tannins Content

Condensed tannin content was measured using a modified vanillin assay method (Julkunen-Tiitto & Sorsa, 2001) [39]. For this assay, OLT extracts were freshly dissolved in distilled water at 1 mg/mL. A 5 μL sample was added to 150 μL vanillin (4%) in methanol and 75 μL HCl (12 N), and the reaction was incubated in the dark for 20 min at room temperature. The absorbance was recorded at 490 nm using a spectrometer. Results were expressed as mg catechin equivalent (CE) per gram of dry sample weight using a (+)-catechin hydrate standard curve (0–1000 μg/mL).

### 2.7. Determination of Proanthocyanins Content

The proanthocyanidin content was quantified using a modified Bate-Smith assay (Porter et al., 1985) [40]. For this assay, OLT extracts were freshly dissolved in distilled water at 1 mg/mL. Briefly, a 25 μL sample was mixed with 300 μL of a n-butanol-HCl-ferric ammonium sulfate (10%) solution (83:6:1), heated to 95 °C for 40 min, and then cooled down. The absorbance was measured at 550 nm using a spectrophotometer. Results were expressed as mg cyanidin chloride equivalents (CCE) per gram of dry sample weight using a cyanidin chloride standard curve (0–500 μg/mL).

### 2.8. HPLC Analysis of Phytochemicals

High-performance liquid chromatography (HPLC) was used to analyze catechins and anthocyanidins, following an adapted method from Ling et al., (2005) [41]. Samples (1 mg/mL in 0.1% phosphoric acid) were filtered through a 0.45 µm membrane. Analysis was performed using a Shimadzu SCL-LC 10A HPLC (Shimadzu, Kyoto, Japan) with a UV-VIS detector (Shimazu, Kyoto, Japan). The separations were performed using a C18 reverse-phase column, Hypersil ODS reverse-phase column (ThermoFisher Scientific, GA, USA; 5 µm, 250 × 46 mm i.d.) for total catechins; Hypersil GOLD (ThermoFisher Scientific, GA, USA; 5 µm, 250 × 4.6 mm i.d.) for OLT chemical compositions (the catechins, caffeine and gallic acid).

### 2.9. Specific Conditions

Total Catechins: A mobile phase of 1% acetic acid (solvent A) and acetonitrile (solvent B) were used, with a linear gradient from A/B (92:8) to A/B (73:27) over 40 min at a flow rate of 1 mL/min. The detection was at 280 nm.

OLT extract composition: A mobile phase of aqueous 0.1% formic acid (solvent A) and 0.1% formic acid in acetonitrile (solvent B) was used, with the leaner gradient elution (Table 1) over 45 min at a flow rate of 1 mL/min. The detection was at 280 nm.

### 2.10. Effect of OLT Extract on NLRP3 Inflammasome Activation

The J774A.1 cells were cultured in RPMI-1640 medium, supplemented with 10% FBS and 2 mM L-glutamine, then maintained in a humidified CO_2_ incubator (MCO-230AIC, PHCbi, Tokyo, Japan) at 37 °C with 5% CO_2_ and regularly passaged. The cells (2  ×  10^6^ in 2 mL of medium) were subjected to two treatment protocols: (1) For the priming stage: Cells were pre-incubated with or without OLT extract with increasing concentrations (0, 25, 50, and 100 μg/mL) for 1 h, followed by the addition of LPS (1 μg/mL final concentration) or saline for 6 h. (2) For the activation stage: Cells were pre-incubated with or without OLT extract with increasing concentrations (0, 25, 50, and 100 μg/mL) or saline for 1 h, then incubated with LPS (1 μg/mL) or saline for 6 h, then washed with saline, and lastly, further incubated with ATP (5 mM) or saline for an additional 30 min. In both assays, the concentrations of IL-6, TNF-α, and IL-1β in the culture medium were quantified using an ELISA kit according to the manufacturer’s instructions (R&D Systems, Minneapolis, MN, USA). Cellular levels of NLRP3 inflammasome protein, activated caspase-1, and pro-caspase-1 were analyzed by Western blotting.

### 2.11. Determination of ASC Oligomerization

Cells were stimulated under the specified conditions for the activation stage (treatment protocol 2) with or without OLT treatment (100 μg/mL). After incubation, cell pellets were collected, washed with TBS and cross-linked with 2 mM DSS for 45 min at 37 °C. Cells were lysed in CHAPS buffer, and lysates were centrifuged sequentially to isolate ASC oligomers. The resulting pellets were resuspended in loading buffer, heated briefly at 90 °C, and stored at −20 °C if needed. ASC oligomerization was then analyzed by Western blotting on a 15% SDS-PAGE. ASC speck formation was further confirmed by fluorescence microscopy (AxioObserver Z1, Zeiss, Oberkochen, Germany).

### 2.12. Determination of ROS Levels

Reactive oxygen species (ROS) production was assessed using DCFH-DA. J774A.1 macrophages were pretreated with oolong tea (OLT) extract at 0, 25, 50, and 100 μg/mL for 1 h. Cells were then stimulated with 1 μg/mL LPS for 6 h. After LPS treatment, cells were incubated with 2 µM DCFH-DA for 30 min. Subsequently, 5 mM ATP was added to activate the inflammasome. ROS fluorescence was recorded every 10 min for 60 min at an excitation wavelength of 485 nm and an emission wavelength of 530 nm using a microplate reader (BioTek Epoch Microplate Spectrophotometer, Agilent Technologies, Santa Clara, CA, USA).

### 2.13. Statistical Analysis

Data are shown as mean ± standard deviation (SD). Statistical significance was determined by two-tailed t-tests for two groups or ANOVA with Dunnett’s multiple comparisons test for three or more groups. *p* < 0.05 were considered to be statistically significant.

## 3. Results

### 3.1. Analysis of Bioactive Phytochemicals from OLT Extract

To characterize the major components in the OLT extract, assays were conducted to measure the total phenolic, flavonoid, condensed tannin, and proanthocyanidin contents. To provide a representative composition of traditionally consumed oolong tea, the OLT extract was analyzed for its major phytochemical groups, including phenolics, flavonoids, condensed tannins, and proanthocyanidins [36].

As shown in Table 2, the total phenolic content was determined to be 321.95 ± 10.58 mg gallic acid equivalents per gram (GAE/g). The total flavonoid content was measured at 64.82 ± 0.83 mg catechin equivalents per gram (CE/g). Additionally, the condensed tannin content was quantified at 233.67 ± 6.61 mg CE/g, while the proanthocyanidin content was 10.88 ± 0.46 mg cyanidin chloride equivalents per gram. These findings indicate that polyphenols are the predominant components in OLT extract, with flavonoids representing a significant secondary group. Table 3 presents the primary catechin compounds and phenolic acid concentrations identified in OLT extract using HPLC analysis. Epigallocatechin gallate (EGCG) was the most abundant compound, with a concentration of 119.97 ± 2.40 µg/mg. Other notable components included epigallocatechin (EGC) at 97.33 ± 0.51 µg/mg and gallic acid at 82.52 ± 1.64 µg/mg. The extract also contained caffeine (32.14 ± 0.59 µg/mg) and catechin (12.98 ± 0.03 µg/mg). These results highlight the diverse bioactive composition of the OLT extract, emphasizing its rich profile of phenolic and flavonoid compounds.

### 3.2. OLT Reduces LPS Priming Outputs (IL-6/TNF-α, pro-IL-1β, NLRP3) While ASC and Pro-Caspase-1 Remain Unchanged

**Figure 2:**
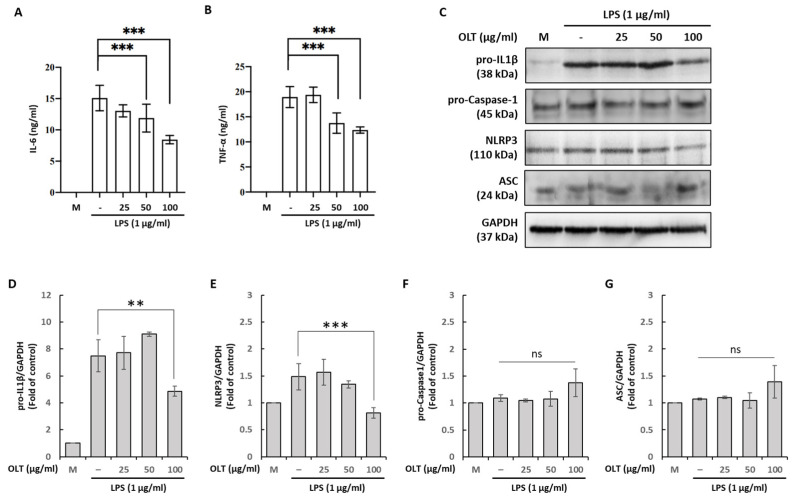
LPS-induced priming stage of macrophages under different OLT treatment conditions (0, 25, 50, and 100 μg/mL). (**A**) IL6 and (**B**) TNF-α were analyzed using ELISA. (**C**) Various priming components were determined with immunoblot analysis, and separately quantified using ELISA (**D**) pro-IL-1β, (**E**) NLRP3, (**F**) ASC and (**G**) pro-Caspase-1. Error bars represent the standard deviation of three independent experiments. ns: non significance, ** and *** represent *p* < 0.01 and *p* < 0.001, respectively.

The activation of the NLRP3 inflammasome involves two steps, priming and activation. To assess the effect of OLT on the priming step in J774A.1 macrophages, cells were pre-treated with OLT (0, 25, 50, 100 μg/mL) prior to LPS stimulation. OLT reduced the secretion of IL-6 and TNF-α in a dose-dependent manner, with 100 μg/mL decreasing IL-6 and TNF-α by ~40% and ~30%, respectively (Figure 2A,B). Immunoblotting showed that OLT attenuated the LPS-induced increases of pro-IL-1β and NLRP3, with significant reductions at 100 μg/mL (Figure 2C–E; *p* < 0.01 for pro-IL-1β; *p* < 0.001 for NLRP3). By contrast, ASC and pro-caspase-1 remained unchanged across OLT concentrations (Figure 2C,F,G; ns), consistent with their constitutive expression during LPS priming.

### 3.3. OLT Inhibits NLRP3 Inflammasome Activation by Suppressing IL-1β Secretion, Caspase-1 Activation, and ASC Oligomerization

**Figure 3:**
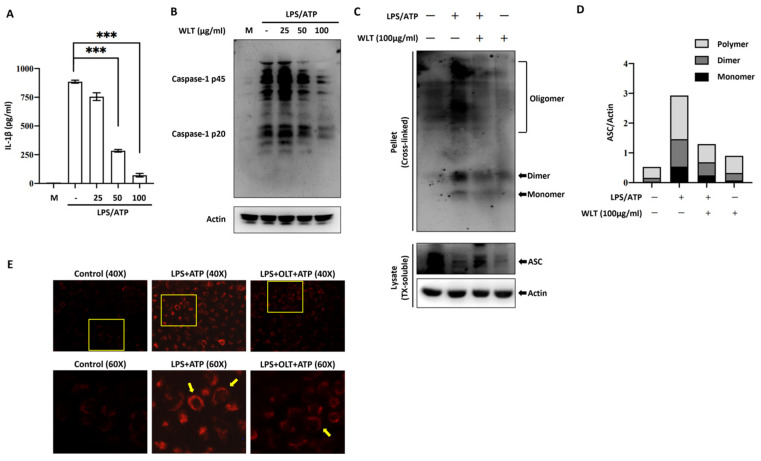
OLT pretreatment suppresses LPS/ATP-induced inflammasome activation in J774A.1 macrophages. (**A**) IL-1β secretion was quantified by ELISA. (**B**) Caspase-1 activation was evaluated in culture supernatants by immunoblotting, showing cleavage from pro-caspase-1 (p45) to the active p20 fragment. (**C**) ASC oligomerization was analyzed by chemical cross-linking and immunoblotting, and (**D**) the relative abundance of ASC polymers, dimers, and monomers was quantified and normalized to actin. (**E**) ASC speck formation was visualized by immunofluorescence microscopy, with representative images shown in red and arrows indicating ASC specks. OLT pretreatment reduced IL-1β release, caspase-1 cleavage, ASC oligomerization, and speck formation in a dose-dependent manner, with the most pronounced inhibition observed at 100 μg/mL. Data are expressed as mean ± SD of three independent experiments. *** indicate *p* < 0.001 versus the LPS/ATP group.

To evaluate the impact of OLT extracts on the activation stage of NLRP3 inflammasome activity in LPS/ATP-stimulated J774A.1 macrophages, we measured IL-1β and Caspase-1 secretion across varying concentrations of OLT (0, 25, 50, and 100 μg/mL). Our results indicate a significant, dose-dependent reduction in IL-1β levels, with the highest concentration of OLT (100 μg/mL) eliciting the most pronounced decrease in IL-1β secretion (Figure 3A). A similar pattern was observed in the expression of Caspase-1 bands. The appearance of the p45 band indicates normal intracellular synthesis of Caspase-1, while the prominent band above p45 corresponds to Pro-Caspase-1, the inactive precursor. The bands around ~33–35 kDa likely represent cleavage intermediates of the activated enzyme in complex with ASC. The p20 band typically denotes the large subunit of active Caspase-1 generated upon inflammasome activation, whereas the ~10 kDa band corresponds to the small subunit. Under high-concentration OLT treatment, all of these Caspase-1 bands were markedly reduced, indicating a significant decrease in their expression levels (Figure 3B).

The modulatory effect of OLT extract (100 μg/mL) on LPS/ATP-induced NLRP3 inflammasome activation was assessed by analyzing ASC oligomerization via immunoblotting and visualizing ASC speck formation through fluorescence microscopy. ASC oligomerization, a key indicator of inflammasome activation, was induced by LPS and ATP treatment. Immunoreactive bands were observed at molecular weights corresponding to ASC monomers, dimers, and larger oligomers (Figure 3C). The additional treatment with OLT extract resulted in a noticeable inhibition of ASC oligomerization as seen in Figure 3C,D. Similarly, OLT pretreatment markedly reduced ASC speck formation under LPS/ATP-stimulated conditions, as shown in Figure 3E. Quantification revealed that 100 μg/mL OLT decreased the proportion of speck-positive cells by more than 50% compared to the LPS/ATP group. Fluorescence microscopy images further confirmed this effect, showing abundant and prominent ASC specks in LPS/ATP-treated cells, whereas OLT-treated cells displayed a marked reduction in visible speck formation.

### 3.4. OLT Extracts Attenuate LPS/ATP-Induced ROS Generation in a Time- and Dose-Dependent Manner

**Figure 4:**
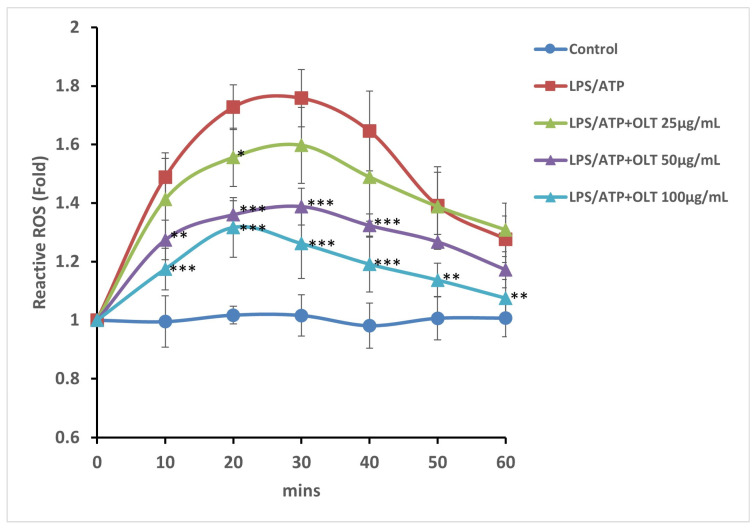
ROS levels induced by LPS/ATP stimulation over 60 min under varying concentrations of OLT extract treatment (0, 25, 50, and 100 μg/mL) and a control group without treatment interference. *, **, and *** indicate *p* < 0.05, *p* < 0.01, and *p* < 0.001 versus the control group.

To evaluate the effects of OLT pretreatment on intracellular ROS generation under LPS/ATP-stimulated conditions, ROS production was monitored over 60 min (Figure 4). In the LPS/ATP group (positive control), ROS levels rapidly increased and peaked at approximately 1.8-fold at 20–30 min, followed by a gradual decline to ~1.4-fold by 60 min. Pretreatment with OLT extract (25–100 μg/mL) resulted in a dose-dependent attenuation of ROS production, while maintaining a similar temporal pattern. The highest concentration (100 μg/mL) showed the most pronounced inhibition, with a peak of ~1.3-fold and a return close to baseline levels by 60 min, approaching those of non-stimulated control cells.

## 4. Discussion

The findings of this study underscore the bioactive potential of oolong tea (OLT) extract, with particular emphasis on its anti-inflammatory effect through modulation of the NLRP3 inflammasome pathway.

Chemical analysis of the OLT extract revealed high levels of total phenolics and condensed tannins in OLT and relatively moderate to less level of total flavonoids and Proanthocyanidins. These compounds have various health benefits, and reported anti-inflammatory effects, suggesting OLT’s potential role in mitigating inflammation [13,53,54,55,56,57]. It also contains a diverse range of bioactive compounds, with the catechins being particularly notable (Table 3). EGCG, a key catechin, detected in high concentrations, is reported to demonstrate therapeutic potential across various diseases, including its anti-inflammatory properties [58]. As we summarized in Table 3, several studies also highlight EGCG’s inhibitory effect on NLRP3 inflammasome activation. Moreover, epicatechin, caffeine, and gallic acid, have also reported to show promise in suppressing NLRP3 activation. This diverse composition highlights OLT’s potential for managing oxidative stress, inflammation, and related health concerns, emphasizing its overall health-promoting properties [59].

While individual compounds in OLT (e.g., EGCG) have been widely studied for effects on NLRP3 inflammasome activity, the potential synergy within the whole beverage is often overlooked. Our data indicate that consuming OLT as an extract modulates both the priming and activation stages of the pathway. During priming, OLT reduced IL-6 and TNF-α in a dose-dependent manner and significantly lowered pro-IL-1β and NLRP3 protein levels, whereas ASC and pro-caspase-1 were unchanged. Upon LPS/ATP stimulation, OLT further decreased IL-1β release (~50% at 100 µg/mL) and attenuated caspase-1 cleavage as well as ASC oligomerization/speck formation. Taken together, these findings support that OLT dampens upstream priming outputs and downstream activation readouts of the NLRP3 inflammasome, highlighting its translational potential as a whole-beverage intervention.

A key mechanism observed in this study is OLT’s disruption of ASC oligomerization and assembly, which are pivotal for inflammasome function. During the priming stage, the formation of ASC specks was significantly reduced, particularly at a concentration of 100 μg/mL. This disruption in ASC assembly led to a cascade effect, impairing the formation of the NLRP3-ASC-Caspase-1 complex, a crucial step for inflammasome activation. The resulting decrease in Caspase-1 activity further dampened the release of IL-1β. The reduction in ASC synergy indicates that OLT interferes with the oligomerization process of the inflammasome, thereby impairing its assembly efficiency and ultimately attenuating the overall inflammatory response. These findings provide additional insights into how OLT modulates the activation stage, highlighting its potential for therapeutic intervention. The significant inhibition of IL-1β secretion and Caspase-1 activation further underscores OLT’s potent effect during the activation stage, positioning it as a promising candidate for targeting inflammasome-mediated inflammatory responses and attenuating the progression of chronic inflammatory diseases [60,61]. Additionally, OLT significantly reduced ASC oligomerization and speck formation, critical steps for inflammasome function. This suggests a broader immunomodulatory potential, as OLT’s actions parallel those observed in catechin treatments that modulate macrophage-mediated inflammatory responses. OLT pretreatment reduced intracellular ROS levels, a key mediator of oxidative stress and inflammasome activation, in a dose-dependent manner. The lowest concentration (25 μg/mL) showed minimal suppression of LPS/ATP-induced ROS production, whereas 50 and 100 μg/mL OLT significantly attenuated the ROS surge, with peak inhibition observed at 20–30 min. The highest concentration (100 μg/mL) exhibited the most pronounced effect, lowering ROS levels to ~1.3-fold and approaching baseline by 60 min. This ROS-scavenging effect is consistent with previous studies showing that tea polyphenols reduce ROS accumulation and lipid peroxidation in LPS-induced inflammatory models [23].

Collectively, these findings suggest that OLT modulates both the priming and activation phases of the NLRP3 inflammasome pathway, effectively suppressing pro-inflammatory cytokines, inflammasome components, and ROS. This comprehensive modulation underscores OLT’s potential as a therapeutic candidate for managing inflammation-driven disorders, including atherosclerosis, type 2 diabetes, and neuroinflammatory diseases. Given that dysregulated NLRP3 activation is implicated in various conditions, such as autoimmune disorders, metabolic syndromes, and chronic inflammatory diseases like Alzheimer’s, OLT’s polyphenolic compounds offer significant promise as natural inhibitors of the NLRP3 pathway, aiding in inflammation control [2,62,63].

In addition, the relationship between ROS reduction and suppression of NLRP3 activation deserves further attention. Our findings indicate that OLT significantly decreases intracellular ROS levels in parallel with attenuated inflammasome activity. Although this correlation is strong, it does not establish direct causality. Previous studies have demonstrated that ROS act as key upstream mediators of NLRP3 inflammasome activation, and that antioxidant treatments can markedly reduce IL-1β release and caspase-1 activation in macrophages [64,65]. Based on this evidence, it is likely that ROS scavenging plays a central role in the anti-inflammatory effects of OLT, although additional mechanisms, such as modulation of ASC oligomerization and mitochondrial signaling, may also contribute. Future studies employing selective ROS modulators will be valuable to definitively establish this causal link.

Furthermore, OLT extract provides comparable benefits to well-known anti-inflammatory phytochemicals like curcumin and resveratrol, owing to its unique blend of polyphenols, catechins, and condensed tannins. By directly inhibiting both inflammasome assembly and cytokine priming, OLT offers a complementary mechanism of action. Notably, we propose that the combined action of these bioactive compounds maximizes OLT’s overall therapeutic potential, surpassing the effects of individual components alone. Its widespread availability as a commonly consumed beverage further enhances its appeal as a natural, convenient anti-inflammatory agent, warranting further investigation.

Another important point concerns the cellular uptake of OLT phytochemicals. Although our study focused on downstream signaling effects, previous reports indicate that catechins and related polyphenols can cross cell membranes primarily through passive diffusion, with additional regulation by efflux transporters such as P-glycoprotein and MRPs [66,67]. Once inside the cell, these compounds can exert antioxidant and anti-inflammatory actions by modulating intracellular ROS and signaling pathways [68]. While the precise uptake dynamics of OLT extract were not addressed in this study, these mechanisms suggest plausible routes for the observed intracellular effects. Future investigations combining uptake assays and transporter inhibition studies will be valuable to clarify how OLT phytochemicals enter macrophages and modulate inflammasome responses.

ASC oligomerization was evaluated to investigate the inhibitory effect of OLT extract on NLRP3 inflammasome activation, demonstrating a significant reduction in inflammasome assembly. While these results highlight the efficacy of OLT in modulating inflammatory responses, certain limitations should be acknowledged. The concentration range of OLT polyphenols required for in vivo efficacy remains to be clearly defined, and variations in brewing methods may influence the bioavailability and activity of these compounds. Future research should include clinical trials to assess the therapeutic potential of OLT in human populations, particularly for inflammatory and autoimmune diseases. Moreover, examining dose–response relationships and the long-term safety of OLT consumption will be essential for validating its clinical applicability.

We acknowledge certain limitations of our study. First, additional structural information could be obtained through Nuclear Magnetic Resonance (NMR) analysis. Whereas we did not use NMR spectroscopy in complex mixtures due to the complexity of the extract and potential limited sensitivity of NMR for low-abundance compounds, future studies may incorporate NMR as a complementary tool for deeper structural elucidation. Next, our study did not directly assess the role of dynein in NLRP3 inflammasome activation. Although we examined downstream markers such as ASC speck formation and oligomerization, further dynein-mediated transport may contribute to upstream inflammasome assembly dynamics and interaction to clarify the cytoskeletal regulation of inflammasome signaling. The omission of DAPI staining in the fluorescence imaging experiments represents another limitation. Although nuclear staining would have aided in visual orientation and normalization, the fluorescence images were acquired with proper focus and consistent imaging conditions. Importantly, the observed differences in signal intensity between treatment groups remain clearly distinguishable and biologically interpretable, even in the absence of DAPI. Moreover, we did not assess IL-18 levels, which are also downstream of inflammasome activation. While IL-1β and ASC oligomerization were used as primary readouts, future studies need to investigate IL-18 to provide a more comprehensive profile of inflammasome-related cytokines.

## 5. Conclusions

This study demonstrates that OLT extract, rich in polyphenols, flavonoids, condensed tannins, and proanthocyanidins, exerts notable anti-inflammatory and antioxidant effects in LPS-stimulated macrophages. OLT effectively reduced intracellular ROS levels and inhibited key components of the NLRP3 inflammasome pathway at both the priming and activation stages. Specifically, OLT downregulated IL-6, TNF-α, pro-IL-1β, and NLRP3 expression in a dose-dependent manner during priming. Upon LPS/ATP stimulation, OLT further suppressed IL-1β secretion, caspase-1 cleavage, ASC oligomerization and speck formation. Collectively, these findings suggest that OLT extract inhibits both upstream signaling and downstream activation of the NLRP3 inflammasome. Further in vivo, clinical and epidemiological studies are warranted to validate its therapeutic potential and safety profile.

## Figures and Tables

**Table 1:** Linear gradient for Oolong tea extraction.

Time (min)	Solvent A (%)	Solvent B (%)
0	90	10
10	90	10
24	80	20
30	78	22
35	75	25
35.1	90	10
45	90	10

**Table 2:** The total content of phenolics, flavonoids, condensed tannins and proanthocyanidins in OLT extract.

Phytochemical Compounds	Content
Total phenolics (mg GAE/g DW)	321.95 ± 10.58
Total flavonoids (mg CTE/g DW)	64.82 ± 0.83
Condensed tannins (mg CE/g DW)	233.67 ± 6.61
Proanthocyanidins (mg CCE/g DW)	10.88 ± 0.46

Notes: mean ± standard deviation (SD). Abbreviations: mg GAE/g DW, mg gallic acid equivalents per g of dry weight; mg CTE/g DW, mg catechin tannin equivalents per g of dry weight; mg CE/g DW, mg catechin equivalents per g of dry weight; mg CCE/g DW, mg cyanidin chloride equivalents per g of dry weight.

**Table 3:** Chemical composition of OLT extract and the potential NLRP3 interactions of each compound.

Compounds	Structure	Molecular Formula	Molecular Weight (g/mol)	Extract Content (µg/mg)	mmol/g	NLRP3-Related Publications
Gallic acid (GA)	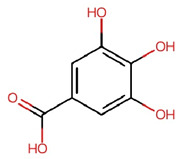	C_7_H_6_O_5_	170.12	4.28 ± 0.02	0.025	Lin et al., 2020 [42] Yu et al., 2023 [43]
Gallocatechin (GC)	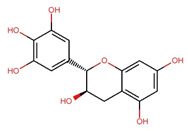	C_15_H_14_O_7_	306.27	61.64 ± 0.30	0.201	–
Epigallocatechin (EGC)	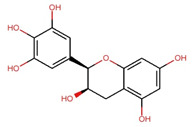	C_15_H_14_O_7_	306.27	97.33 ± 0.51	0.318	–
Catechin (C)	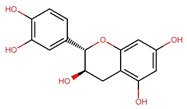	C_15_H_14_O_6_	290.27	12.98 ± 0.03	0.045	–
Caffeine	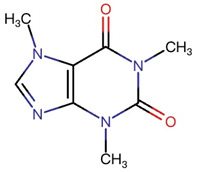	C_8_H_10_N_4_O_2_	194.19	32.14 ± 0.59	0.166	Zhao et al., 2019 [44] Wang et al., 2022 [45]
Epicatechin (EC)	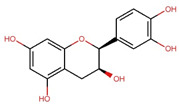	C_15_H_14_O_6_	290.27	17.27 ± 0.09	0.059	Tian et al., 2021 [46] Wu et al., 2022 [47]
Epigallocatechin gallate (EGCG)	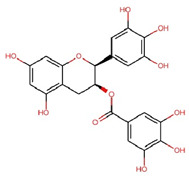	C_22_H_18_O_11_	458.4	119.97 ± 2.40	0.262	Abundant related research and articles can be found; here are a selected few: Jhang et al., 2016 [48] Di et al., 2022 [49] Gao et al., 2016 [50] Lee et al., 2019 [51] Wang et al., 2020 [52]
Gallocatechin gallate (GCG)	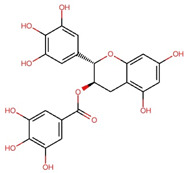	C_22_H_18_O_11_	458.4	82.52 ± 1.64	0.180	–
Epicatechin gallate (ECG)	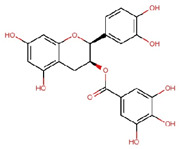	C_22_H_18_O_10_	442.4	17.92 ± 0.38	0.041	–
Catechin gallate (CG)	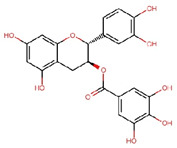	C_22_H_18_O_10_	442.4	2.90 ± 0.18	0.007	–

Notes: mean ± standard deviation (SD). Abbreviations: Chemical analysis of the OLT extract identified a complex mixture and diverse array of compounds, including a variety of active catechins: (+)-catechin (C), (−)-gallocatechin (GC), (−)-epigallocatechin (EGC), (−)-epicatechin (EC), (−)-epigallocatechin gallate (EGCG), (−)-gallocatechin gallate (GCG), (−)-epicatechin gallate (ECG), (−)-catechin gallate (CG), the major alkaloid caffeine and the main tea phenolic acid, gallic acid (GA). Values are presented as mean ± SD (*n* = 3). All chemical structures were drawn and modified from the Research Collaboratory for Structural Bioinformatics Protein Data Bank—Chemical Sketch Tool.

## Data Availability

The original contributions presented in this study are included in the article. Further inquiries can be directed at the corresponding author.
